# Severe Cerebral Small Vessel Disease Burden Is Associated With Poor Outcomes After Endovascular Thrombectomy in Acute Ischemic Stroke With Large Vessel Occlusion

**DOI:** 10.7759/cureus.13122

**Published:** 2021-02-04

**Authors:** Destiny Hooper, Tariq Nisar, David McCane, Jason Lee, Ken Chyuan Ling, Farhaan Vahidy, Kelvin Wong, Stephen Wong, David Chiu, Rajan Gadhia

**Affiliations:** 1 Neurology, Houston Methodist Hospital, Houston, USA; 2 Statistics, Houston Methodist Neurological Institute, Houston, USA; 3 T.T. and W.F. Chao Center for BRAIN, Houston Methodist Neurological Institute, Houston, USA

**Keywords:** leukoaraiosis, cerebral small vessel disease, large vessel occlusion, acute ischemic stroke, endovascular thrombectomy, recanalization

## Abstract

Background

Despite recent advancements in the treatment of acute ischemic stroke (AIS) with large vessel occlusion (LVO), infarct progression over time and functional outcomes remain variable. This variation in outcomes may be partially attributed to an underlying state of chronic cerebral hypoperfusion and ischemia affecting small cerebral perforating arterioles, venules, and capillaries of the brain; broadly termed cerebral small vessel disease (CSVD). We investigated the association between CSVD burden and the degree of disability following successful recanalization with endovascular thrombectomy (EVT) in patients with AIS presenting with LVO.

Methodology

We conducted a single center retrospective analysis of all patients presenting with AIS LVO between May 2016 and May 2019. Patients who were premorbidly independent and presented within six hours from the last known well (LKW) with a proximal anterior circulation occlusion confirmed on computed tomography (CT) angiography of the head or neck were treated with EVT. Patients presenting after six hours and up to 24 hours from LKW with a target ischemic core to perfusion mismatch profile on CT or magnetic resonance (MR) perfusion, or a clinical imaging mismatch on MR diffusion-weighted imaging, were also treated. Patients with successful revascularization, defined as a thrombolysis in cerebral infarction score 2b or 3, were included and evaluated for CSVD burden. The presence of CSVD was quantified using the Fazekas scale (0-3). All patients were further evaluated for disability at 90 days using the modified Rankin Scale (mRS, range 0-6). An mRS score of ≤2 was defined as a good functional outcome.

Results

Of the 190 patients evaluated, absent (Fazekas grade 0), mild (Fazekas grade 1), moderate (Fazekas grade 2), and severe (Fazekas grade 3) CSVD was present in 33 (17.4%), 84 (44.2%), 35 (18.4%), and 38 (20.0%) patients, respectively. Patients with severe CSVD (Fazekas grade 3) were found to be older, had a higher presenting National Institute of Health Stroke Scale (NIHSS), and had greater proportions of preexisting atrial fibrillation and dementia compared to patients with no CSVD (Fazekas grade 0). Using a multivariate ordinal logistic regression model to adjust for age, presenting NIHSS, thrombus location, LKW to groin puncture time, use of tissue plasminogen activator, ischemic infarct volume, development of a symptomatic intracerebral hemorrhage, and treatment with hemicraniectomy, patients with Fazekas grade 3 were significantly more likely to have poor 90-day functional outcomes compared to patients with Fazekas grade 0 (odds ratio 10.25, 95% confidence interval [3.3-31.84]).

Conclusions

Based on our analytical cohort of AIS LVO patients treated with EVT, we found that patients with severe CSVD burden had worse functional outcomes at 90 days and increased mortality. These results provide evidence that the burden of CSVD may be considered an independent risk factor of poor clinical outcome and a predictor of mortality in patients with AIS presenting with LVO, despite successful radiographic recanalization with EVT.

## Introduction

Acute ischemic stroke (AIS) with large vessel occlusion (LVO) is an acute vascular emergency that is often clinically devastating. Until recently, intravenous tissue plasminogen activator (tPA) was the only approved treatment for AIS patients, inclusive of the most severe ischemic stroke subtype, LVO. Following a series of randomized clinical trials demonstrating a significant improvement in functional outcomes in select patient populations, recanalization with endovascular thrombectomy (EVT) has become the standard of care in AIS LVO [1-3]. However, despite continued advancements in the treatment of LVO, infarct progression over time remains variable, with functional outcomes ranging from complete recovery to death, with varying degrees of disability in between [1]. Because of the variability in outcomes, it would be of clinical value to identify patients who fail to improve with endovascular intervention prior to the initiation of treatment, limiting potentially futile recanalization procedures.

It has been hypothesized that the heterogeneity in outcomes observed post-EVT may be in part attributed to the underlying state of chronic cerebral hypoperfusion and ischemia affecting small perforating cerebral arterioles, venules, and capillaries, currently referred to as cerebral small vessel disease (CSVD). Prolonged hypoperfusion, often associated with advanced age, uncontrolled hypertension, and smoking, has been shown to cause endothelial dysfunction and failure of these vessels. This results in reduced micro-vessel density, deficient collateral flow, and ultimately, a reduced functional reserve in the brain’s most metabolically active nuclei and complex white matter networks [4]. These pathological changes, characterized by patchy to confluent white matter lesions in the periventricular and subcortical areas of the brain, are a common nonspecific imaging finding that was first described on neuroimaging by Fazekas et al. in 1987 [5]. The burden of these white matter hyperintensities (WMH), as seen on magnetic resonance imaging (MRI), has previously been shown to correlate with worse functional outcomes in patients with AIS, including those treated with tPA [6,7]. Additionally, greater WMH burden has been associated with an increased incidence of intracerebral hemorrhage (ICH) in patients following recanalization [8-10]. Thus, a better understanding of the relationship between CSVD severity and functional outcomes in LVO becomes increasingly important, as it may help mitigate outcome differences in patients undergoing EVT and could serve as a guide for targeted and personalized treatment strategies.

In this study, we aimed to further evaluate the potential influence of CSVD burden on long-term functional outcomes in AIS LVO patients undergoing EVT. We hypothesized that a higher CSVD burden would correlate with worse 90-day functional outcomes after adjusting for other clinically important co-variants.

This article was previously presented as a meeting abstract at the 2020 European Stroke Organization and World Stroke Organization Conference on November 7, 2020 and won the Best Poster Award in the topic “Prognosis.”

## Materials and methods

**Figure 1:**
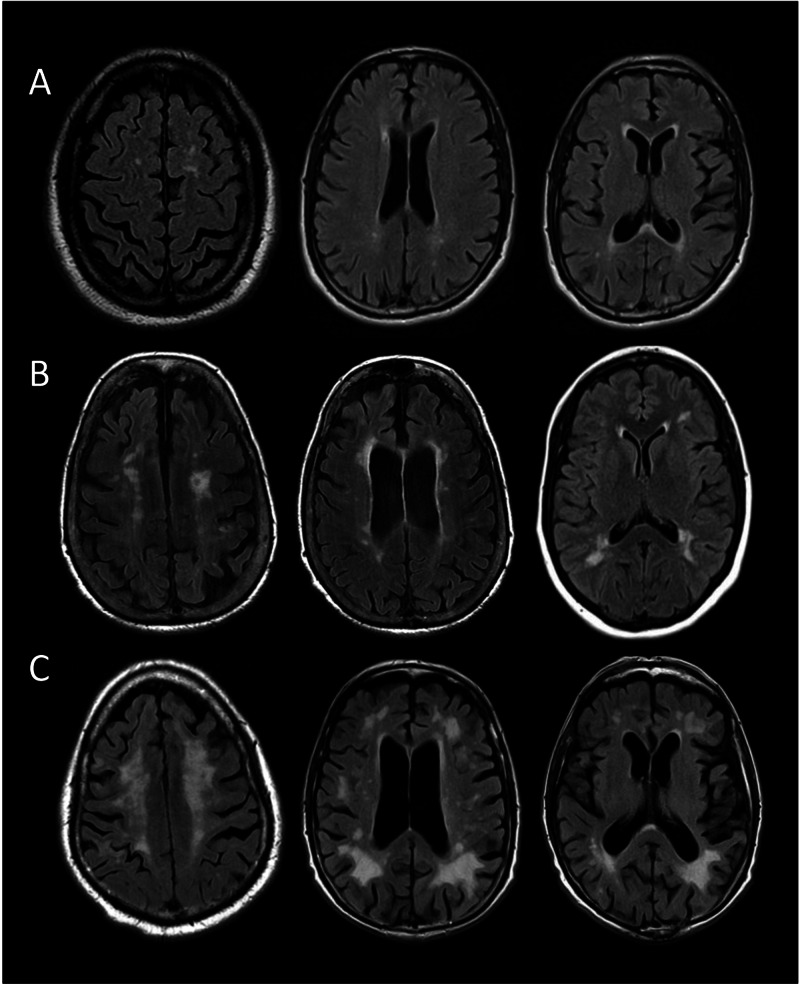
Grading of leukoaraiosis severity based on the Fazekas scale. (A) Fazekas grade 1 (mild) with periventricular caps of the ventricles or punctate foci in the deep white matter; (B) Fazekas grade 2 (moderate) with smooth periventricular halo or convergence of deep white matter lesions; (C) Fazekas grade 3 (severe) with confluent periventricular leukoaraiosis extending into the subcortical deep white matter. MRI examples from actual patient population. MRI = magnetic resonance imaging

We conducted a single center retrospective analysis of all patients presenting with AIS LVO between May 2016 and May 2019. Patients who were premorbidly independent and presented within six hours from last known well (LKW), defined as the patient being witnessed at baseline, with a proximal anterior circulation occlusion (internal carotid artery, M1 or proximal M2 segment of middle cerebral artery) confirmed on computed tomography angiography (CTA) of the head or neck were treated with EVT. Patients presenting in the extended window time frame, after six hours and up to 24 hours from LKW, with a target ischemic core to perfusion mismatch profile on CT or magnetic resonance (MR) perfusion (ischemic core volume <70 cc, mismatch ratio >1.8, and mismatch volume >15 cc) or a clinical imaging mismatch on MR diffusion-weighted imaging (DWI) (<20 core infarct, NIHSS ≥10, age ≥80 years or <30 cc core infarct, NIHSS ≥10, age <80 years or 31-50 cc core infarct, NIHSS ≥20, age <80 years) were also included [2,3]. EVT was performed with stent retrievers (Solitaire stent-Ev3), thromboaspiration (Penumbra Ace61), or a combination technique according to the preference of the on-call neurointerventionalist. The degree of recanalization achieved following thrombus removal was defined using the thrombolysis in cerebral infarction (TICI) grading system. Patients with >50% reperfusion in the vascular distribution of the occluded artery, TICI score 2b or 3, were included and evaluated for CSVD burden. The presence of CSVD was defined as supratentorial periventricular and deep subcortical WMH in the asymptomatic, nonischemic hemisphere, as visualized on a 3.0 T MRI unit using axial T2-fluid-attenuated inversion recovery (FLAIR) sequences to avoid the confounding effect of the acute ischemic lesion. Patients who were unable to undergo MRI due to medical instability, implantable devices, or death or those with bilateral ischemic infarcts were excluded. The severity of CSVD was quantified using the Fazekas scale (0-3), with grade 0 indicating no periventricular or deep WMH, grade 1 defined as periventricular caps or pencil-thin lining of the ventricles or punctate foci in the deep white matter, grade 2 defined as a smooth periventricular halo or convergence of deep white matter foci in the subcortical regions, and grade 3 representing severe, confluent periventricular leukoaraiosis extending into the subcortical deep white matter. Examples of different grades are depicted in Figure 1. Each region was assigned a grade by a neurology-trained physician blinded to patient outcomes. The periventricular and subcortical grades were then averaged and a single Fazekas grade reflecting the cumulative CSVD burden was assigned for all patients. All patients were further assessed by a stroke research nurse, in-person or through a telephone interview, at 90 days using the modified Rankin Scale (mRS, range 0-6), to determine the degree of long-term disability. Patients who received a score of ≤2 were determined to have a good functional outcome.

Additional factors were also evaluated to assess for possible confounding variables; age, sex, preexisting comorbidities including hypertension, hyperlipidemia, diabetes mellitus, coronary artery disease, atrial fibrillation, prior stroke, chronic kidney disease, and dementia, as well as the presenting National Institute of Health Stroke Scale (NIHSS) assessed by a stroke-trained physician, thrombus location (ICA, M1, M2), LKW to groin puncture time, the use of tPA per the AHA/ASA guidelines, ischemic infarct volume, symptomatic ICH defined as any sign of hemorrhage on follow-up CT head and a clinical deterioration of four points or greater from the initial NIHSS, and emergent treatment with hemicraniectomy. Patient demographics, clinical characteristics, and 90-day mRS implemented in the study were obtained using the prospective Houston Methodist Hospital Outcomes-Based Prospective Endpoints in Stroke (HOPES) registry [11]. This study protocol was reviewed and approved by the Houston Methodist Hospital Institutional Review Board. Ischemic infarct volumes were calculated using a fully automatic segmentation deep learning algorithm developed at the Houston Methodist Neurological Institute. MR DWI (b = 1000 s/mm^2^), exponential apparent diffusion coefficient (eADC) maps were used as inputs, and ischemic infarct volumes were defined as a hyperintense lesion on DWI with an eADC correlate below the irreversible threshold of ADC <620 × 10^-6^ mm^2^/s. All segmented infarct volumes were verified manually by the developer of the algorithm.

Statistical Methods

Descriptive analyses were implemented to summarize continuous variables using distributionally appropriate measures of central tendency and spread, that is, mean ± standard deviations (SD) and median with interquantile range (IQR). Univariable analyses, utilizing t-test, Wilcoxon rank-sum test, and chi-square test, were conducted to compare characteristics of patients with varying levels of CSVD burden. A pairwise Wilcoxon test, analysis of variance, and Kruskal-Wallis tests were also used to identify differences between the continuous variables in patients with different degrees of CSVD burden. A multivariate ordinal logistic regression model with shift analysis was used to estimate the independent association of CSVD burden (Fazekas scale 0-3) with 90-day functional outcomes (mRS 0-6) among AIS LVO patients. The dependent variable (90-day mRS) and the primary independent variable (Fazekas scale) were both modeled as ordinal variables. The odds ratios (OR) and 95% confidence intervals (CI) were reported, providing likelihood estimates of a shift to higher mRS for a given comparison between grade 0 (reference category) and subsequently higher Fazekas grades. Proportionality of odds assumption and the overall fit of the models were assessed utilizing standard published approaches. All analyses were performed using R statistical software (version 3.6.1).

## Results

A total of 329 AIS LVO patients were screened, of which 273 had a confirmed anterior circulation LVO and achieved successful recanalization with EVT. Of these patients, 83 were unable to undergo MRI or were excluded for lack of documented metrics. In the remaining 190 patients, absent (Fazekas grade 0), mild (Fazekas grade 1), moderate (Fazekas grade 2), and severe (Fazekas grade 3) CSVD was present in 33 (17.4%), 84 (44.2%), 35 (18.4%), and 38 (20.0%) patients, respectively. There was an even distribution of both genders across the spectrum of Fazekas grades (p < 0.05); however, patients in the Fazekas grade 3 category were significantly older compared to patients with grade 0 (81 vs. 52 years, p < 0.001). Furthermore, comorbidities were similarly distributed across all of the Fazekas grades, with the exception of atrial fibrillation and dementia, which had a higher proportion in patients with Fazekas grade 3 WMH burden compared to grade 0 (68% vs. 15%, 18% vs. 0%). The median NIHSS was also found to be higher in patients with Fazekas grade 3 compared to grade 0 (16 vs. 9; p < 0.001). Baseline characteristics of the included 190 patients, stratified by Fazekas grades, are summarized in Table 1.

**Table 1:** Baseline characteristics of patients undergoing mechanical thrombectomy stratified by CSVD burden measured by the Fazekas score. SD = standard deviation; LDL = low density lipoprotein; Hb = hemoglobin; NIHSS = National Institute of Health Stroke Scale; tPA = tissue plasminogen factor; LKW = last known well; TICI = thrombolysis in cerebral infarction; mRS = modified Rankin Score; CSVD = cerebral small vessel disease

Characteristics	Fazekas 0 (n = 33)	Fazekas 1 (n = 84)	Fazekas 2 (n = 35)	Fazekas 3 (n = 38)	P-Value
Age (years) (mean, SD)	51.58 ± 14.18	69.07 ± 11.95	75.49 ± 11.41	80.66 ± 10.7	<0.001*
Gender					
Female (%)	39.39	51.19	48.57	68.42	0.094
Male (%)	60.61	48.81	51.43	31.58	0.094
Comorbidities					
Hypertension (%)	93.94	88.10	88.57	97.37	0.339
Hyperlipidemia (%)	87.88	88.10	91.43	84.21	0.826
LDL cholesterol (mean, SD)	111.81 ± 34.42	95.96 ± 31.93	100 ± 45.89	82.95 ± 32.18	0.010*
Diabetes mellitus (%)	33.33	38.10	25.71	39.47	0.564
HbA1c (mean, SD)	6.32 ± 1.75	6.33 ± 1.72	6.02 ± 1.38	6.41 ± 1.26	0.450
Atrial fibrillation (%)	15.15	50.00	54.29	68.42	<0.001*
Coronary artery disease (%)	18.18	21.43	25.71	31.58	0.536
Prior stroke (%)	24.24	21.43	20.00	21.05	0.978
Chronic kidney disease (%)	9.09	22.62	22.86	26.32	0.300
Dementia (%)	0.00	5.95	8.57	18.42	0.028*
Metrics					
NIHSS (median, quantile)	9 [25% = 8, 75% = 17]	17 [25% = 10.75, 75% = 22.25]	19 [25% = 14.5, 75% = 24.5]	16 [25% = 10.5, 75% = 20.75]	<0.001*
Received tPA (%)	42.42	46.43	37.14	39.47	0.781
LKW to puncture (min) (mean, SD)	443.55 ± 364.97	376.56 ± 359.54	329.11 ± 306.14	486.26 ± 676.62	0.500
TICI - 2b (%)	36.36	40.48	28.57	34.21	0.657
TICI - 2c (%)	0.00	0.00	0.00	2.63	0.259
TICI - 3 (%)	63.64	59.52	71.43	63.16	0.680
Infarct size (cc) (mean, SD)	56.18 ± 105.14	69.79 ± 95.49	63.11 ± 78.98	56.05 ± 68.36	0.830
Intracerebral hemorrhage (%)	9.09	5.95	5.71	10.53	0.781
Decompressive hemicraniectomy (%)	6.06	8.00	2.86	2.63	0.523
Outcomes					
mRS 0 (%)	33.33	14.00	8.57	0.00	<0.001*
mRS 1 (%)	24.24	25.00	14.29	2.63	0.020*
mRS 2 (%)	12.12	13.00	8.57	2.63	0.330
mRS 3 (%)	3.03	7.14	20.00	13.16	0.077
mRS 4 (%)	3.03	13.10	14.29	18.42	0.260
mRS 5 (%)	15.15	7.14	14.00	29.00	0.017*
mRS 6 (%)	9.09	20.24	20.00	34.21	0.077

**Figure 2:**
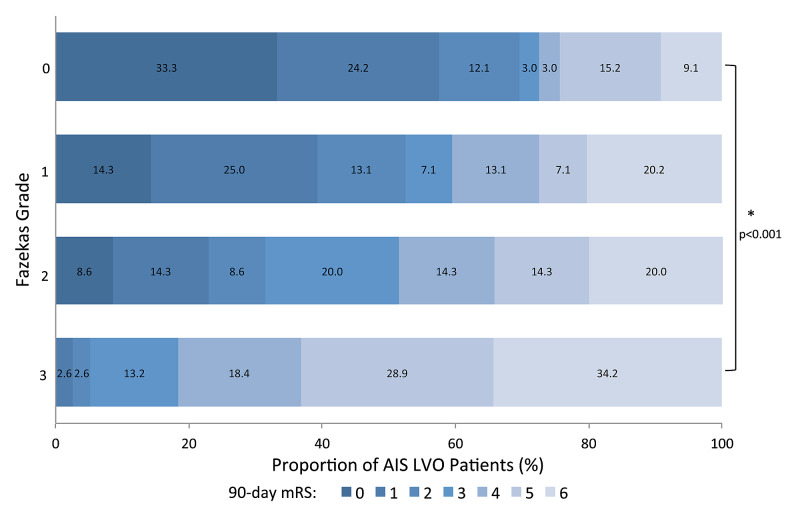
Outcome distribution per CSVD severity as quantified by the Fazekas scale. CSVD = cerebral small vessel disease

Using a multivariate ordinal logistic regression model to adjust for age, presenting NIHSS, LKW to groin puncture time, thrombus location, use of tPA, ischemic infarct volume, development of symptomatic ICH, and intervention with hemicraniectomy, a higher likelihood of a poor outcome (mRS shift to the right) was observed for all comparisons (Figure 2). This shift towards a higher mRS was not found to be statistically significant among patients with Fazekas grade 1 or 2 compared to grade 0. However, patients with Fazekas grade 3 were approximately 10 times more likely to have poor functional outcomes at 90 days, with the majority of these patients suffering from severe disability compared to patients with grade 0 (OR = 10.25, 95% CI [3.3-31.84], p < 0.001). These results are detailed in Table 2. Additionally, no difference in mortality was found in patients with Fazekas grade 1 or 2 compared to grade 0 (OR = 2.54, 95% CI [0.69-9.31], p = 0.161; OR = 2.50, 95% CI [0.59-10.63], p = 0.215). Conversely, patients with Fazekas grade 3 had a five-fold increased risk of mortality at 90 days compared to grade 0 (OR = 5.2, 95% CI [1.33-20.32], p = 0.018). Despite worse functional outcomes and increased mortality rates in patients with Fazekas grade 3, no increased incidence of sICH was observed in this patient population. However, it is important to note that patients with poor functional outcomes (mRS ≥ 3) were found to have larger ischemic infarct volumes (99.43 cc ± 107.11 cc vs. 19.06 cc ± 32.01 cc, p < 0.001) and an increased incidence of sICH (13.10% vs. 8.55%, p < 0.05) compared to patients with good functional outcomes (mRS ≤ 2) overall.

**Table 2:** Odds of a poor neurological outcome (mRS score ≥ 3). OR = odds ratio; CI = confidence interval; mRS = modified Rankin Score

Variable	OR	95% CI	P-Value
Fazekas grade 0	Reference	Reference	Reference
Fazekas grade 1	1.26	0.52-3.02	0.610
Fazekas grade 2	2.00	0.67-5.98	0.214
Fazekas grade 3	10.25	3.3-31.84	<0.001*

## Discussion

Based on our cohort of AIS LVO patients who were successfully recanalized with EVT, the presence of severe CSVD, as graded by the Fazekas scale on MRI T2-FLAIR, was found to correlate with worse functional outcomes at 90 days. Furthermore, the severity of CSVD was also found to be associated with increased mortality in this patient population. These findings were independent of gender, preexisting vascular risk factors, LKW to groin puncture time, thrombus location, or concurrent use of tPA. These results can likely be attributed to reduced micro-vessel density, deficient collateral blood flow, and poor cerebrovascular reserve in patients with greater CSVD burden. Thus, these pathological changes impede the brain’s innate resistance to ischemia, resulting in rapid infarct progression following vessel occlusion, despite timely and successful recanalization [12-14].

These results provide further evidence to the already established literature reporting an association between severe CSVD and worse functional outcomes in patients with AIS treated with thrombolysis [6-10]. However, there are only a few retrospective studies that have evaluated this association in patients with AIS LVO post-EVT [15-20]. Of these, the majority reported comparable findings, demonstrating a strong association between the severity of CSVD burden and long-term disability. However, the effect of severe CSVD burden on mortality, following recanalization with EVT, has been less supported.

While some discrepancies exist across the current literature, the unique strengths of this study include a clinically heterogeneous and socio-demographically diverse patient population, the use of quantitative MRI as the imaging modality, application of the Fazekas scale for visual assessment of leukoaraiosis, calculation of infarct volume using novel automated software, procedural success in all included subjects, and the inclusion of patients who presented with LVO in the extended window time frame, up to 24 hours from LKW, which we felt was a critical patient population to study. It is important to note that the majority of previous studies did not include patients presenting beyond eight hours from LKW. Additionally, compared to previous studies, MRI was the main imaging modality utilized in this study as CT is both less sensitive and less specific to chronic WMH compared to MRI T2-FLAIR, particularly in patients with mild leukoaraiosis [21]. It should also be acknowledged that recent studies have suggested that leukoaraiosis may confound the interpretation of CT perfusion and thus bias patient eligibility for extended window EVT due to an overestimation of the hypoperfused area [22]. Thus, further investigation of CSVD burden in patients excluded from treatment with EVT in the extended time window is warranted. Additionally, we used the Fazekas scale for its well-established accuracy in quantifying leukoaraiosis on MRI in patients with ischemic stroke compared to other visual rating scales more appropriate for defining WMH in demyelinating diseases and dementia [23]. Furthermore, we restricted our analysis to patients who achieved complete recanalization with a TICI score of 2b or 3 to avoid potential confounding of clinical outcomes by radiographically failed procedures.

Additionally, previous studies have demonstrated a strong association between severe CSVD and hemorrhagic transformation following intervention with both tPA and EVT, which was believed to be a consequence of endothelial dysfunction and blood vessel fragility in these patients [8-10,24-26]. Conversely, we found no association between CSVD burden and the incidence of symptomatic ICH post-EVT. One proposed explanation for this discrepancy may be the use of varying definitions of ICH implemented in different studies. In our study, symptomatic ICH was defined as a type 2 parenchymal hemorrhage with a deterioration in NIHSS of ≥4 points or death [27].

There were also limitations in this study that must be acknowledged. First, this was a retrospective analysis from a single center registry with a relatively small sample size. Second, we chose to only include patients on whom we could consistently and accurately quantify CSVD burden using radiological parameters. Thus, several patients were excluded due to their inability to undergo MRI, particularly those with permanent pacemakers. This patient population deserves further evaluation. Furthermore, CSVD burden was assessed using a visual scale as opposed to a more precise volumetric calculation, creating a potential for bias. Lastly, the status of leptomeningeal collaterals was not graded precluding an analysis of their influence on functional outcomes.

Despite these limitations, we present a carefully conducted analysis of a well-characterized cohort of 190 AIS LVO patients who underwent standardized protocol for EVT and achieved successful recanalization. Our analysis significantly contributes to the growing body of literature on this topic and provides further evidence that CSVD burden may be an independent risk factor of poor long-term prognosis and mortality in patients with AIS presenting with LVO, despite successful revascularization with EVT [28].

## Conclusions

Several predictors of favorable clinical outcomes in AIS LVO treated with EVT have been reported, both in the acute and extended time window. However, further validated radiographic biomarkers are needed to guide future evidence-based treatment strategies. The implementation of CSVD burden as an imaging marker for the brain’s vascular reserve, to be used alone or in combination with already established quantitative imaging scores, may serve as a pertinent independent predictor of EVT success. Unfortunately, at this time, not enough data exist to influence therapeutic decision-making in patients with AIS LVO who are eligible for EVT as per the current guidelines. Further studies, in the form of large randomized controlled clinical trials, are needed to better understand the underlying mechanism of CSVD and its relationship with functional outcomes in LVO.
